# Sugar Alcohols Have a Key Role in Pathogenesis of Chronic Liver Disease and Hepatocellular Carcinoma in Whole Blood and Liver Tissues

**DOI:** 10.3390/cancers12020484

**Published:** 2020-02-19

**Authors:** Israa T. Ismail, Oliver Fiehn, Ashraf Elfert, Marwa Helal, Ibrahim Salama, Hala El-Said

**Affiliations:** 1 National Liver Institute, Menoufia University, Shebeen El Kom 55955, Egypt; imismail@ucdavis.edu (I.T.I.); ashraf.yousif@liver.menofia.edu.eg (A.E.); marwa.helal@liver.menofia.edu.eg (M.H.); ibrahiem.a.alqader11@liver.menofia.edu.eg (I.S.); hala.elsaid@liver.menofia.edu.eg (H.E.-S.); 2 NIH West Coast Metabolomics Center, University of California Davis, Davis, CA 95616, USA

**Keywords:** sorbitol, cirrhosis, carbohydrates, amino acids, nucleosides, microbial metabolism, microbiota, cancer

## Abstract

The major risk factors for hepatocellular carcinoma (HCC) are hepatitis C and B viral infections that proceed to Chronic Liver Disease (CLD). Yet, the early diagnosis and treatment of HCC are challenging because the pathogenesis of HCC is not fully defined. To better understand the onset and development of HCC, untargeted GC-TOF MS metabolomics data were acquired from resected human HCC tissues and their paired non-tumor hepatic tissues (*n* = 46). Blood samples of the same HCC subjects (*n* = 23) were compared to CLD (*n* = 15) and healthy control (*n* = 15) blood samples. The participants were recruited from the National Liver Institute in Egypt. The GC-TOF MS data yielded 194 structurally annotated compounds. The most strikingly significant alteration was found for the class of sugar alcohols that were up-regulated in blood of HCC patients compared to CLD subjects (*p* < 2.4 × 10^−12^) and CLD compared to healthy controls (*p* = 4.1 × 10^−7^). In HCC tissues, sugar alcohols were the most significant (*p* < 1 × 10^−6^) class differentiating resected HCC tissues from non-malignant hepatic tissues for all HCC patients. Alteration of sugar alcohol levels in liver tissues also defined early-stage HCC from their paired non-malignant hepatic tissues (*p* = 2.7 × 10^−6^). In blood, sugar alcohols differentiated HCC from CLD subjects with an ROC-curve of 0.875 compared to 0.685 for the classic HCC biomarker alpha-fetoprotein. Blood sugar alcohol levels steadily increased from healthy controls to CLD to early stages of HCC and finally, to late-stage HCC patients. The increase in sugar alcohol levels indicates a role of aldo-keto reductases in the pathogenesis of HCC, possibly opening novel diagnostic and therapeutic options after in-depth validation.

## 1. Introduction

Hepatocellular carcinoma (HCC) represents about 75% of all liver cancers [1]. Worldwide, liver cancers belong to the three most common causes of cancer-related deaths [2,3]. The disparity in HCC prevalence in different areas of the world matches the geographical distribution of risk factors [4]. Hepatitis B (HBV), hepatitis C (HCV), alcohol and non-alcoholic fatty liver disease are the main risk factors of chronic liver disease (CLD) [5,6]. CLD often progresses to liver cirrhosis, a major risk factor for HCC. HCV and HBV together with HCC itself have an increasing incidence in the USA [7], China [8] and Africa [9]. Egypt has the highest incidence of chronic hepatitis C and HCC in the Middle East [10,11]. Subtle manifestation of HCC in its early stages is responsible for its delayed diagnosis and poor prognosis [12]. The laboratory diagnosis of HCC by alpha-fetoprotein (AFP) lacks sensitivity and specificity with respect to CLD and cirrhotic liver [13,14]. Early HCC can be treated by liver resections or other treatments, but such options may not be available for cases with a late-stage diagnosis of HCC [15]. Multiple studies outlined the pathogenesis of HCC in relation to risk factors [16,17,18]. However, on the molecular and metabolic level, the pathogenesis of HCC and its progression from CLD and cirrhosis is not well understood [19].

The liver plays a central role in metabolism and directly impacts levels of many blood metabolites. Hence, HCC pathogenesis in the tissue should be readily diagnosable by blood metabolomics [15]. Profiling substrates, cofactors, and products of a large diversity of biochemical pathways lead to a better understanding of disease etiologies, diagnosis, prevention, and, possibly, treatment [20]. Previous metabolomics studies have been conducted on serum, plasma, urine or tissues of HCC patients using mass spectrometry [21,22] or nuclear magnetic resonance (NMR) [23,24]. HCC pathogenesis induced alterations in levels of amino acids [25], lipids [21], bile acids [26] and redox state metabolism [24,27,28,29]. Gas chromatography/mass spectrometry (GC/MS) provided good coverage of primary metabolism in HCC ranging from energy metabolism [14] to glycolysis, TCA metabolites, purines, and lipids [30,31]. Specifically, GC/MS revealed alterations in amino acids, sugars, fatty acids, and other macromolecules in biofluids of HCC patients [22,25,32,33,34,35,36]. However, none of these earlier studies combined analyses of both tissues and biofluids for detailing the pathogenesis of HCC in patients. We, therefore, focused on the impact of chronic liver disease and hepatocellular carcinoma on primary metabolism in both liver and blood. Specifically, we aimed to (1) detail alterations in primary metabolism defined by the progression of viral CLD to HCC in comparison to healthy subjects and (2) to validate these metabolomic differences in resected human hepatocellular carcinoma compared to paired non-malignant hepatic tissues.

## 2. Results

### 2.1. Characteristics of Subjects

23 HCC and 15 CLD patients with HCV or/and HBV infection were recruited in addition to 15 healthy control subjects. All CLD patients had underlying current viral infections. All HCC patients had either current viral infection diagnosed by PCR at the time of surgery or a previous infection ranging up to 2 years prior to surgery. The diagnosis of hepatocellular carcinoma was confirmed by histopathologic studies after resection. The characteristics of the study subjects are provided in Table 1. Subjects were well matched, showing no significant differences in age (using ANOVA test) and gender (using the Chi-square test) between HCC, CLD, and healthy control subjects. In addition, there were no significant differences in aspartate transaminase (AST), alanine transaminase (ALT), and platelet count between CLD and HCC groups (using *t*-test). Similarly, current positive PCR detection of HCV and HBV infection did not show significant differences between CLD and HCC groups (using the Chi-square test) (Table 1), excluding these potentially confounding effects. As expected, the HCC group showed a significant increased Alfa fetoprotein (AFP) level compared to CLD patients (*p* = 0.02), as well as prothrombin time expressed as an international normalized ratio (INR) (*p* = 0.005) and decreased level of hemoglobin (*p* = 0.011). 

As commonly observed [37,38,39], HCC cases were dominated by men, with only 26% of the cases presented by women (Table 2). Single focal lesions represented 65% of the cases, and lesions <5 cm in size represented 83% of the cases. HCC paired tissue samples were subdivided into two subgroups (Table 2): 16 tissues were classified as early-stage HCC cases (diagnosed as stage I and II) versus 7 cases of late-stage HCC patients (diagnosed as stage III, IV).

### 2.2. Statistical Metabolic Classes Differentiating HCC, CLD, and Healthy Control Blood Samples

Metabolomics data from 53 blood samples (23 HCC patients, 15 CLD patients, and 15 healthy controls) were acquired by GC/MS analysis and BinBase data processing for compound identification [40]. Instead of using classic GC/quadrupole mass spectrometry, we employed GC-time-of-flight MS to double the number of identified metabolites in comparison to previously published studies [21,22,23,24,25,26,27,28,29]. A total of 730 GC-MS peaks were detected of which 194 compounds were reported as identified chemical structures using authentic reference standards. Peak intensities were normalized to total metabolome abundance, and data were statistically assessed by multivariate and univariate tests.

**Figure 1:**
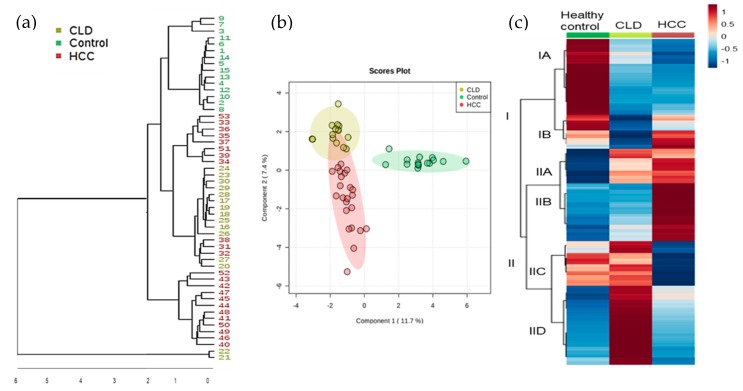
Multivariate clustering of blood-based metabolomics data using GC-TOF mass spectrometry. (**a**) Unsupervised hierarchical clustering of all 730 detected metabolites in the studied groups HCC, Chronic Liver Disease (CLD), and healthy control. (**b**) Supervised sparse partial least squares-discriminant analysis of all 730 metabolites to better distinguish metabotypes of study groups. (**c**) Clustering of 510 significant metabolites (Mann–Whitney U test *p* < 0.05 and qFDR < 0.1) across the three test groups, range-scaled from increased (+1, red) to decreased (−1, blue).

We first conducted an unsupervised hierarchical cluster analysis of all 730 GC-MS peaks (Figure 1a) that yielded three main clusters, dividing all healthy subjects from CLD and HCC patients. Two CLD cases were found as clear outliers. Yet, these two cases remained in all subsequent statistical analyses because there were no physiological or pathological differences that warranted excluding these cases. Two dendrogram clusters consisted solely of HCC cases or were mixed between HCC and CLD cases (Figure 1a). We did not find discernible clinical phenotypes such as late-stage versus early-stage HCC that could explain the metabolic difference between these clusters. Next, we performed supervised multivariate statistics to learn whether metabolic signatures were strong enough to discriminate between CLD and HCC patients. Figure 1b plots the results of a sparse Partial Least Squares-Discriminant Analysis (sPLS-DA). The plot confirms that the largest difference between the groups were among healthy versus diseased subjects. Interestingly, the former two CLD outliers were now correctly grouped to the CLD subjects, showing that a metabolic signature exists that distinguishes CLD from HCC patients. Yet, supervised clustering may suffer from statistically overfitting and hence, such results need to be confirmed in subsequent follow-up studies before using specific metabolites for a clinical diagnostic test. To test for specific metabolites that distinguished CLD versus HCC subjects, we performed a Kruskal–Wallis test and found 510 out of 710 metabolites significantly different between both groups (qFDR < 0.1), including 147 structurally identified metabolites (Supplementary S1). For two-group comparisons including the healthy controls, the Mann-Whitney U test revealed 111 significant compounds (*p* < 0.05; qFDR < 0.10) between healthy control versus CLD patients and 90 significant compounds (*p* < 0.05; qFDR < 0.10) between CLD versus HCC subjects (Supplementary S1).

To understand the magnitude of metabolic differences between healthy subjects, CLD and HCC patients and to find classes of metabolites that distinguished between these groups, we range-scaled differences for all compounds and used the group averages for secondary cluster analysis (Figure 1c). Figure 1c demonstrates significant metabolic differences between the three groups, ranging across different pathways and compound classes. Supplementary S1 details the contribution of each of these significant differences to the two main clusters I and II, defined by the first branch in the heatmap dendrogram. Cluster IA mainly separated healthy controls from both CLD and HCC subjects, whereas Cluster II showed significant differences between CLD and HCC subjects. Specifically, Cluster IIB comprised metabolites that were largely increased in HCC subjects in comparison to both CLD and healthy control subjects. Conversely, Cluster IID consisted of metabolites that were elevated in CLD patients but neither in HCC subjects nor in healthy controls. Few compounds further highlighted differences between CLD and HCC subjects but not in healthy subjects, such as succinic acid from Class IIC. Supplementary S1 details the different compounds found in classes IA-B and IIA-D. Unidentified compounds can be investigated online using the BinVestigate search tool using the corresponding BinBase identifiers [41].

### 2.3. Chemical Set Enrichment Differences Between HCC, CLD, and Healthy Control Blood Samples

#### Metabolomics Data

**Figure 2:**
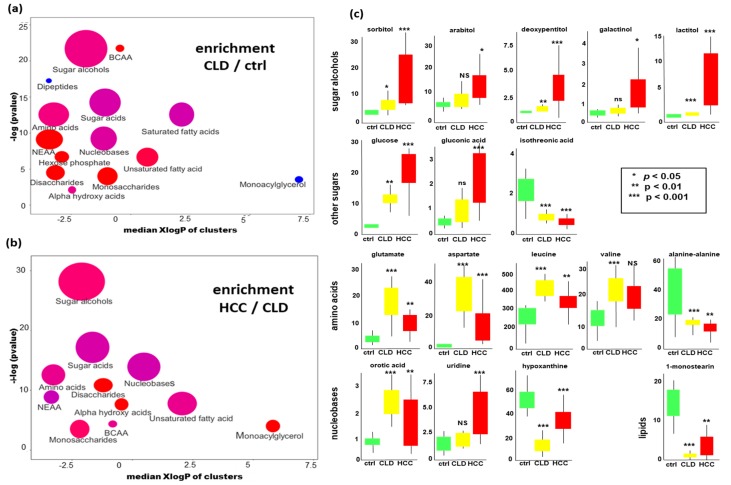
Significant differences in blood metabolites in HCC versus CLD patients and healthy controls. (**a**) Chemical set enrichment statistics (ChemRICH) comparing CLD to healthy control subjects. (**b**) ChemRICH statistics comparing HCC to CLD subjects. Colors of chemical sets: red = increased, blue = decreased, purple = sets with compounds decreased and increased. Ball sizes represent the number of compounds per metabolic set. X-axes represent median lipophilicity (XlogP) of the metabolic clusters. (**c**) Univariate statistics of selected metabolites showing significant differences. Units of all y-axes: normalized peak intensities in 1000 ion counts/spectrum for each quantification ion (Supplementary S1).

Next, we investigated if there were specific groups of metabolites that differed in blood levels between HCC, CLD and healthy control subjects. For this purpose, we performed chemical set enrichment statistics (ChemRICH, [42]). ChemRICH automatically classified all identified compounds in our metabolomic data into 16 chemically similar sets. Unlike to pathway enrichment software (e.g., to KEGG Atlas maps [43]), ChemRICH does not discard any identified metabolite, and it does not consider any compound in more than one set. Classifying compounds into a few sets largely decreases the importance of false discovery rate adjustments in comparison to analyzing all 194 identified compounds in classic univariate statistics. We found 14 significantly altered sets of compounds at *p* < 0.05 when comparing CLD to healthy control subjects (Figure 2a). Specifically, CLD patients showed increased levels of branched-chain amino acids, sugar alcohols, monosaccharides, disaccharides, amino acids, and unsaturated fatty acids. Conversely, dipeptides and monoacylglycerols were found to be decreased in CLD patients compared to healthy controls subjects. Comparing HCC versus CLD patients, we found further increases in sugar alcohols, disaccharides, monosaccharides and amino acids (Figure 2b). Specifically, a range of sugar alcohols were largely increased in HCC subjects with a *p*-value of 2 × 10^−12^ and qFDR of 3.3 × 10^−11^.

To specify which individual compounds in these metabolic classes different between the three study groups, we performed univariate statistics using the Mann-Whitney U test. Full data are given in Supplementary S1, and examples of univariate differences are given in Figure 2c. While most hexitol- and pentitol-dependent blood sugars were found increased in HCC versus CLD patients, the hexitol myo-inositol and several hexitol-containing disaccharides were decreased in this comparison. Other compound classes also showed complex patterns within each metabolic module. For example, while most amino acid levels increased in CLD patients compared to healthy controls (Figure 2c), some amino acids decreased when progressing from CLD to HCC disease states (Figure 2c). 

Compounds related to nucleobase metabolism also showed complex patterns, with increased levels of orotic acid (a breakdown product of UMP) but decreased levels in hypoxanthine (an adenine catabolite) when comparing CLD to healthy control subjects (Figure 2a,c). Similarly, nucleobase metabolism was following distinct patterns when comparing HCC to CLD patients, for example, by exerting a significantly different ratio of the UMP-metabolites uridine/orotic acid (Figure 2b,c). Detailed differences for all compounds are given in Supplementary S1.

### 2.4. Metabolomics of Liver Tissues

**Figure 3:**
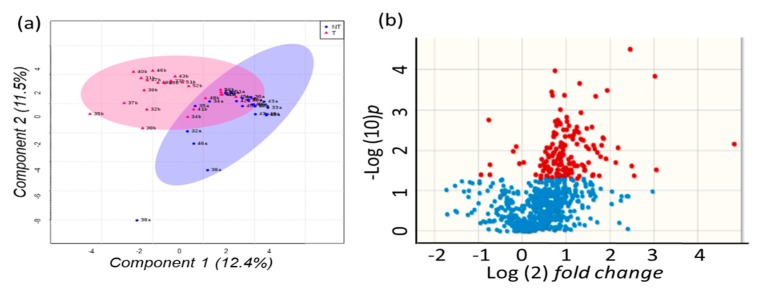
Multivariate analysis of 730 peaks detected by untargeted GC-TOF MS metabolomics analysis of paired HCC tumors versus non-malignant liver tissues. (**a**) sparse Projections to Latent Structures Discriminant Analysis (sPLS-DA). Blue: non-malignant tissues, red: tumor biopsies. Percentages give the total explained variance for vectors 1 and 2. (**b**) Volcano plot for differential regulation of metabolites. Blue: not significant at raw *p* < 0.05, red: significant changes at *p* < 0.05.

Next, we investigated if changes in blood metabolite levels were reflected by changes in resected HCC tumor tissues compared to paired non-malignant surrounding hepatic tissues. 730 unique GC-TOF MS peaks including 194 identified metabolites were used to test for multivariate supervised discriminant analysis (sparse Projections to Latent Structures Discriminant Analysis, sPLS-DA). Figure 3a shows that sPLS-DA discriminates metabolic phenotypes of paired HCC tumors and corresponding non-malignant tissue samples for many patients. However, at least three patient tissues showed only a small variance in this plot and located in the overlapping regions of tumors and non-malignant tissues. The corresponding volcano plot (Figure 3b) showed that around 1/3 of all detected metabolites were found differentially regulated at raw *p* < 0.05 and that most compounds were higher in intensity in tumors than in non-malignant tissues.

**Figure 4:**
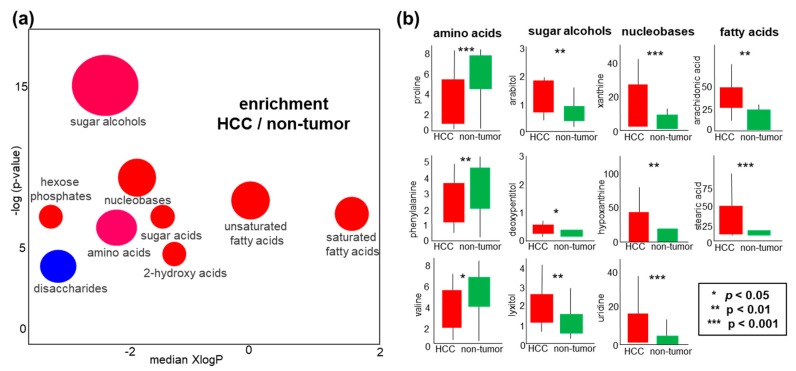
Differential regulation of liver metabolites in HCC tumors versus non-malignant tissues. (**a**) Chemical set enrichment statistics of significantly regulated metabolites. Colors of chemical sets: red = increased, blue = decreased, purple = sets with compounds decreased and increased. Ball sizes represent the number of compounds per metabolic set. XlogP represents the median lipophilicity of the metabolic clusters. (**b**) Examples of boxplots (medians and interquartile ranges) of individual compounds that showed univariate significant differences. Units of all y-axes: normalized peak intensities in 10,000 ion counts/spectrum for each quantification ion (Supplementary S1).

To understand the regulation of metabolic pathways in human liver cancers, we focused on the 64 identified metabolites that showed significant differences in tumors versus non-malignant liver tissues. 39 of these compounds were significant in multiple testing at qFDR < 0.1 (Supplementary S2). All 194 identified metabolites were used to test for significance in ChemRICH chemical set enrichment analysis (Figure 4a). Interestingly, just as in blood analyses, sugar alcohols were found as the most significant chemical set that differentiated HCC malignant tissues from non-malignant tissues (*p* < 1 × 10^−6^). In addition, sugar acids, nucleobases, hexose phosphates, saturated and unsaturated fatty acids were increased in HCC liver tissues. Only disaccharides were found to be decreased in HCC tumors versus non-tumor tissues, for example, trehalose and sophorose (Supplementary 2). Such uncommon metabolites are detected in patients with liver disease due to dysfunction of the intestinal barrier and bacterial overgrowth [44]. Examples of individual compounds are given in boxplots (Figure 4b), showing that specific amino acids such as proline, valine, and phenylalanine were decreased in HCC tumors while most other metabolites were found increased (Figure 4b), including nucleobases that are needed for cell division. Similarly, both saturated and unsaturated free fatty acids were increased (Figure 4b, Supplementary 2).

### 2.5. Sugar Alcohols are Highly Discriminant Metabolites Differentiating HCC from Non-HCC Subjects and HCC-Subjects from CLD Patients

**Figure 5:**
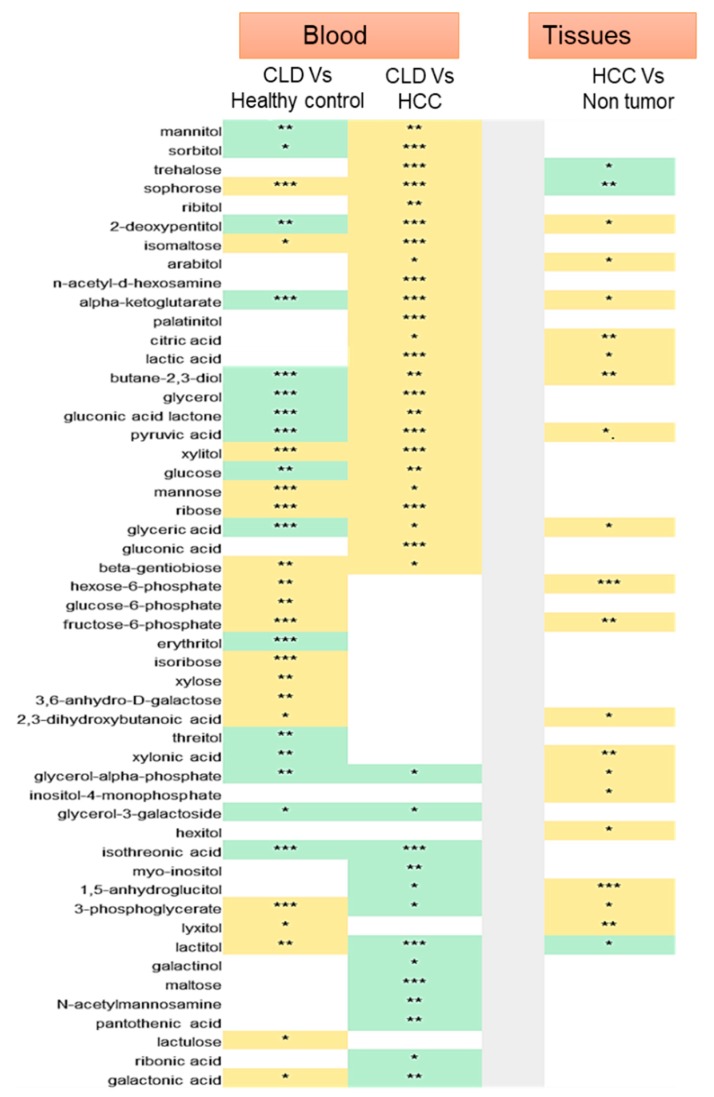
Sugar alcohols and carbohydrate metabolites in Chronic Liver Disease (CLD) and HCC. Heatmap indicating significant differences in blood of CLD versus healthy control (CLD versus healthy controls), HCC versus CLD (HCC Vs CLD) and HCC tissues versus its paired non- malignant hepatic tissues. Non-significant comparisons are left blank. Significant up-regulation yellow, down-regulation green. Stars give significance levels (*** *p* < 0.001, ** *p* < 0.01, * *p* < 0.05). Details in Supplementary S1 and S2.

In blood analyses, we found sugar alcohols as a main metabolic class differentiating HCC from CLD subjects, and CLD from healthy controls (Figure 2a,b). Importantly, we confirmed sugar alcohols also to be the main class differentiating HCC tissues from non-malignant hepatic tissues (Figure 4a). Therefore, we performed detailed analyses of these sugar alcohols and their precursor metabolites (Figure 5). Clinically, the most important question is to distinguish HCC subjects from CLD patients using blood analyses. We, therefore, searched for compounds that were found differentially regulated in blood levels as well as in HCC tumors. Interestingly, we found a huge shift between CLD and HCC subjects for the direct products of aldose reductase, sorbitol, and mannitol. These compounds were significantly down-regulated in CLD but strongly up-regulated in HCC subjects. Interestingly, we found only trends towards a significant increase in sorbitol in HCC tumors versus its paired nontumor hepatic tissues (Supplementary S2). Other sugar alcohols were either up-regulated in all comparisons (such as xylitol) or indicative of liver disease itself such as lactitol that was found heavily upregulated in CLD patient blood compared to healthy controls but downregulated in HCC blood as well as in HCC tumors. Conversely, 2-dexoypentitol was found in opposite directions: it was clearly down-regulated in blood samples of CLD patients, but up-regulated in blood levels of HCC subjects as well as in HCC tumors. The classic tumor-driven primary metabolites citric acid, α-ketoglutarate, pyruvate, and lactate were all significantly up-regulated in HCC tumors as well as in the blood of HCC patients, but down-regulated in the direct comparison of CLD subjects and healthy controls. These examples show that whole blood metabolomic analyses can be very informative in distinguishing patient groups, including compounds that belong to classic tumor metabolism. These examples further show that different types of sugar alcohols show distinct patterns and do not follow a simple explanatory rule. Instead, we noted that a range of compounds may stem from microbial and food metabolism. For example, trehalose and sophorose were found in significantly higher levels in HCC patient blood samples, but at lower concentrations in HCC tumors. These compounds are typical microbial metabolites that may originate from increased intestinal permeability for microbiota and its related metabolites during HCC progression [45]. Other blood metabolites such as maltose, a starch degradation compound, are indicative of liver dysfunction but do not reflect HCC tumor metabolism itself.

**Figure 6:**
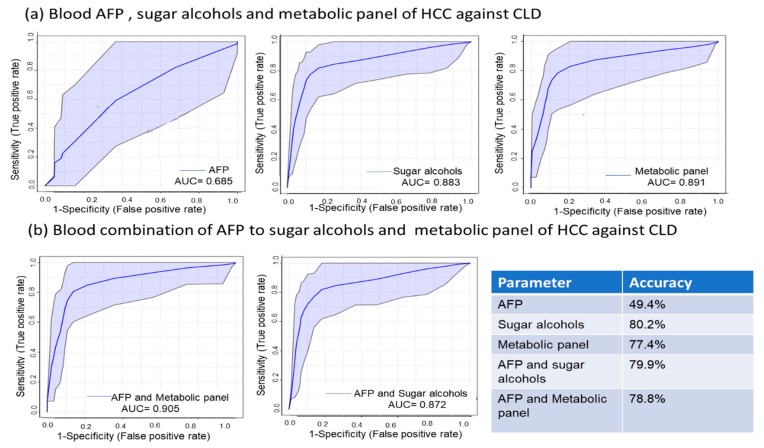
Receiver operating characteristic (ROC) curve analysis of a 6-metabolite panel of discriminating metabolites, a panel of blood sugar alcohols, and AFP to differentiate HCC from chronic liver disease (CLD). (**a**) ROC curve of AFP, 6-metabolite panel, and sugar alcohols to differentiate HCC from CLD and associated accuracy of each comparison. (**b**) ROC curve of combined AFP with the 6-metabolite panel, and sugar alcohols to differentiate HCC from CLD and associated accuracy of each comparison.

Next, we tested how well sets of individual metabolites might better discriminate patient groups than the classic HCC laboratory diagnosis by alpha-fetoprotein, AFP. Here, we first used a panel consisting of only the six most discriminant metabolites between the blood of HCC and CLD patients. With a combination of GC-MS data for maltose, isomaltose, glucose, a-ketoglutarate, lactate and pyruvate, a Receiver Operating Characteristic (ROC) curve and corresponding areas under the curve (AUC) clearly outperformed the classic AFP test (Figure 6). The six-compound panel yielded an AUC of 0.891 with an accuracy of 77.4% while AFP only gave an AUC of 0.685 with an accuracy of 49.4%. When using a panel that only consisted of six discriminating sugar alcohols (sorbitol, arabitol, 2-deoxypentitol, butane-2,3 diol, glycerol-3-galactoside, and lactitol), we received an AUC of 0.883 with an accuracy of 80.2%. When adding the classic AFP marker to those two panels, we did not obtain any significant improvement in AUC or accuracy.

### 2.6. Metabolite Clusters Enable Early Detection of HCC but not Differentiation of HCC Stage Subgroups

**Figure 7:**
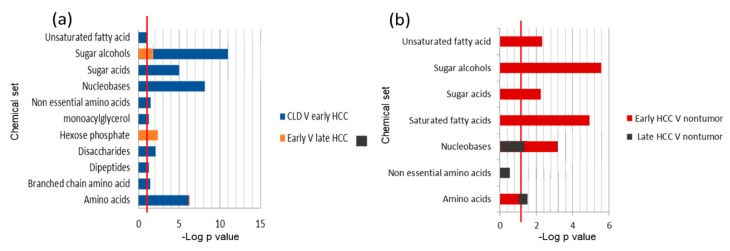
ChemRICH set enrichment statistics comparison for blood (**a**) and HCC tissues metabolites (**b**). (**a**) Significant chemical sets in blood metabolites for early HCC patients versus CLD subjects (blue) and comparing early-stage HCC versus late-stage HCC (orange). (**b**) Significant chemical sets in HCC tumor metabolites to paired non-malignant liver tissues for early HCC stages (red) and late HCC stages (black). The set enrichment significance level of *p* < 0.05 (−log *p* = 1.3) is indicated by red lines.

Last, we stratified subgroups of patients to deepen our understanding of the pathogenesis of the disease on a metabolic level. For blood analyses, we found that early-stage HCC patients (stage I and II) could successfully discriminate the disease metabolic phenotypes from CLD patients using ChemRICH metabolite set enrichment statistics (Figure 7a). Seven metabolite sets showed raw *p*-values <0.05 and nine sets showed qFDR < 0.1 in ChemRICH analyses (Figure 7a and Supplementary S1). Here, sugar alcohols were by far the most discriminant group with *p* = 5.8 × 10^−12^ significance. This analysis showed that ChemRICH discrimination of patient groups in overall blood analysis (Figure 2) was not just based on late-stage HCC patients (stage III and IV). In fact, few blood metabolite sets were different between late-stage and early-stage HCC patients (Figure 7a, Supplementary S1). Specifically, hexose phosphates were differentially regulated between late and early-stage HCC patients (*p* = 0.004, qFDR = 0.07) while sugar alcohols were not significant in qFDR analysis (Supplementary 1). Univariate statistical analysis using the Mann–Whitney U test showed 75 known significant blood metabolites (qFDR < 0.1) when comparing early-stage HCC patients against CLD subjects. When comparing late-stage HCC patients to early-stage HCC patients, we did not find any significant difference at qFDR < 0.1 while at raw *p*-value < 0.05, 20 compounds were significant.

We also analyzed HCC subgroup differences based on tumor metabolism and non-malignant tissues. ChemRICH set enrichment statistics found 5 significant chemical clusters in early HCC hepatic tissues compared to its paired nonmalignant tissues, on the level of both raw-p values and qFDR corrected significance levels. Sugar alcohols were found as the most significant set of metabolites with *p* = 2.7 × 10^−6^ and qFDR = 4.3 × 10^−5^ (Figure 7b and Supplementary S2). In univariate analyses by the Wilcoxon signed rank test we found 26 compounds at qFDR < 0.1 for early HCC stage tumors compared to non-malignant tissue and none for late HCC stage tumors to paired non-malignant tissues (Supplementary S2). In ChemRICH set enrichment statistics, only amino acids showed significant differences at raw *p* = 0.02 but no differences in qFDR correction. These results indicate that for late-stage HCC, the overall hepatic metabolic function was affected, while for early-stage HCC, the surrounding (non-malignant) hepatic tissue was still mostly functioning at a normal level.

## 3. Discussion

While our study was small with respect to the number of patients, we here present the first metabolomics study comparing HCC to CLD and healthy controls that used both blood and tissue samples. We used gas chromatography-time of flight mass spectrometry to study metabolic differences for two main reasons: (a) it is known that tumors largely reprogram primary metabolism. GC-MS is particularly well suited to test for a large variety of primary metabolites in a single-assay analysis. (b) GC-MS instruments are installed in over 100,000 laboratories worldwide, including in many clinical chemistry laboratories. Hence, discriminating metabolites discovered here might be further validated and used in clinical practice without expensive changes in laboratory routines or equipment. We first used multivariate analyses (such as heatmaps, sPLS-DA, and ChemRICH plots) to gain overviews on which metabolic pathways were dysregulated, how well metabolic phenotypes distinguish cases from controls, and how much in-group variance contributed to outlier cases (Figure 1, Figure 2a,b, Figure 3a and Figure 4a). These discovery-driven statistics were followed by classic univariate analyses to attempt finding small panels of (blood-based) discriminatory biomarkers. Such small sets of diagnostic markers could be readily developed into routine clinical assays with classic quadrupole GC-MS instruments and single-ion quantifications using authentic internal standards. The idea is that such panels could be further validated as novel kits in clinical diagnostics for early diagnosis of HCC (Figure 6).

Hepatocellular carcinoma (HCC) is the third most prevalent cause of cancer-related mortality worldwide [46,47]. HCC has various risk factors in different regions in the world [6]. In countries with a high incidence of HCC such as Egypt and China, the primary risk factors are HBV and HCV infections [48]. Chronic HCV infection also continues to be a principal risk factor of HCC in the USA together with non-alcoholic fatty liver disease [46]. HCC manifests with subtle clinical phenotypes that impede HCC diagnosis especially in early stages of the disease [49]. Yet, late diagnosis of HCC correlates negatively with disease outcome prognosis [50]. Hence, we need to establish and validate new assays to better understand HCC pathophysiology and to diagnose HCC in its early stage in comparison to chronic liver disease. Alpha-fetoprotein (AFP) is routinely used as a noninvasive laboratory test, but it has shown poor sensitivity and specificity as a biomarker for HCC [49,51,52]. In accordance with these studies, AFP in our study showed poor power to discriminate HCC from CLD patients. In contrast to these reports, Mehinovic et al. [53] found that AFP is a good biomarker to diagnose HCC patients. AFP specificity is affected by patient sex and race [54] which may partly explain this discrepancy in findings. Moreover, AFP accuracy is higher for HCC in patients without prior HCV infection [55]. Yet, in our study, HCC patients presented with >60% current HCV infection or 91% combined current or prior HCV infection (21/23 patients, Table 1). 

We set out to probe primary metabolism at the level of biochemical pathways and chemical sets by study blood and tissue metabolomics regarding possible pathogenesis and potential diagnostics. Cellular metabolism differs from compounds excreted from cells into the circulatory system. Hence, we designed our study to use both tissues and whole blood instead of serum or plasma, to avoid biases with respect to metabolite detections [56]. Indeed, we found a range of pathway-based metabolites (such as glycolytic intermediates and classic cancer metabolites) that were differentially regulated in whole blood of HCC patients and corresponding HCC tumors. For example, it is well established that many tumor cells consume more glucose than surrounding tissues, converting it to lactate regardless of oxygen availability [57]. This Warburg effect is reflected in our study by detecting increased levels of pyruvate and lactate in both whole blood and HCC tumors. In addition, we confirmed a range of known cancer-related metabolic regulations, for example, increased citrate levels in blood and HCC tumors. Citrate is the substrate of citrate lyase-dependent formation of acetyl-CoA for fatty acid metabolism inside the cells. Similarly, we found a range of further altered cancer metabolic pathways, from fatty acids to nucleotides and proteinogenic amino acids [58]. The increase of these fatty acids in HCC tissues compared to paired non-malignant hepatic tissues is consistent with earlier studies [59,60,61], confirming aberrant lipid metabolism in HCC tissues. Fatty acids are important for cancer cell proliferation [62]. Similarly, we found important differences in nitrogen metabolism, specifically branched-chain amino acids and non-essential amino acids. Our results confirm earlier studies reporting increased blood levels in these amino acids in CLD patients and subsequent decreased levels in HCC patients [63]. These metabolic shifts from CLD to HCC can be explained by an increased expression of branched-chain amino acid enzymes to enhance tumor proliferation and may reflect chemotherapy resistance [63,64,65]. Non-essential amino acids such as aspartate and proline have diverse functions in cancer metabolism, including as antioxidants in redox status metabolism but also as substrates for post-translational and epigenetic modifications [66].

The liver is the primary organ for the metabolism of carbohydrates, specifically to maintain glucose homeostasis through glycogenolysis and gluconeogenesis [67]. Accordingly, large alterations in monosaccharides, disaccharides, and hexose phosphates were detected in our study and confirmed in earlier studies [68,69,70,71,72,73]. In tumor cells, a high glycolytic flux spills over into adjacent metabolic pathways such as the pentose phosphate and hexsoamine pathways [74] which may explain the activation of alternative pathways for glucose metabolism such as the production of sugar alcohols [75]. Sugar alcohols are derived through the polyol pathway [76]. The principle reaction of this pathway is converting glucose to sorbitol by aldo-keto reductases, catalyzed by a group of reductases including aldehyde reductases (EC 1.1.1.2) and aldose reductases (EC 1.1. 1.21). Subsequently, sorbitol may convert to fructose by sorbitol dehydrogenase [77]. A similar spill-over effect of glycolysis has been established as an ancillary pathway in type 2 diabetes mellitus [77,78,79,80]. Increased activity of the polyol pathway was also reported in other liver diseases such as Wilson’s disease that is characterized by copper accumulation in the liver and other organs [81]. Activation of the polyol pathway was also reported in ovarian and cervical cancer cell lines [82,83], lung tumors [75], and colon [75,84], breast, ovary, cervix, and rectum cancers [85]. Recently, the sugar alcohol maltitol was studied as imaging contrast agent in brain tumors [86]. In liver diseases, dysregulation of sugar alcohols was reported in mice and liver cell lines in fatty liver [87] and in mouse cell lines induced hepatoma [88,89,90,91].

The enzymes of the polyol pathway are not expressed in all organs, and the specific physiological roles of the pathway are not yet well understood [82]. For example, expression of the aldo-keto reductase enzyme superfamily [92] disappeared from fetal liver and brain by the 16th week of gestation while sorbitol dehydrogenase expression continued to increase [93]. In rat hepatoma, the role of sugar alcohols was suggested to detoxify harmful aldehydes into inactive alcohols [90]. Gene expression of aldose reductase was increased in rats livers during the development of hereditary hepatitis and hepatoma with aging [88]. This finding was also observed in cancerous lesions compared to non-pathogenic surrounding liver regions [94]. Overexpression of mRNA for aldo-keto reductases was detected in HCC tissues compared to minimal expression in non-malignant liver tissues [95]. The authors interpreted this finding as detoxification of aldehydes by immortal cancer cells to render them resistant to cytotoxic drugs [88,94,95]. 

The functional role of aldo-keto reductases in cancer metabolism is not fully determined [96,97,98,99,100]. This reductase superfamily is associated with oxidative stress and inflammation initiated carcinogenesis [96]. The consumption of cellular NADPH by aldo-keto reductases reduces its availability for the regeneration of the main antioxidant glutathione (GSH), hence, may weaken intracellular antioxidant defenses [101]. The role of aldo-keto reductases in the mediation of cell signals initiated by cytokines, growth factors and chemokines has been also demonstrated in tumorigenesis [102]. In addition, inhibition of aldo-keto reductases prevents epidermal- and fibroblast growth factor-induced migration and invasion of cancer cells [103].

**Figure 8:**
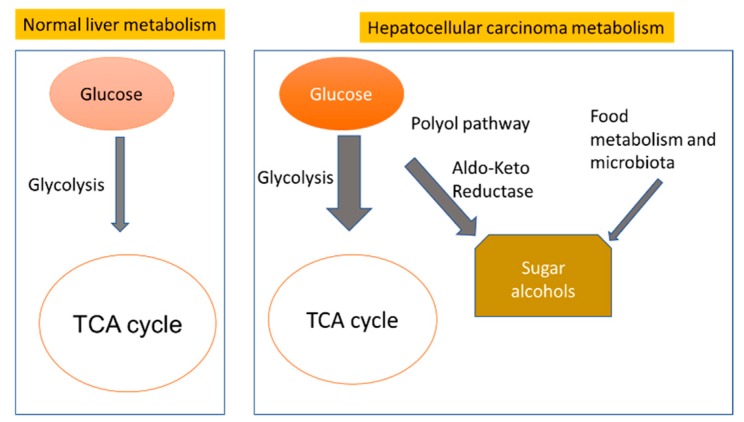
Alternative pathways of carbohydrate metabolism in normal liver metabolism, and dysregulation in hepatocellular carcinomas.

Glycolysis, gluconeogenesis, glycogen metabolism and the tricarboxylic acid cycle (TCA) are the main metabolic pathways in the healthy liver to control normal glucose homeostasis in the human body (Figure 8). Liver diseases, including HCC, lead to dysregulation of glucose homeostasis [104] and activate minor pathways such as polyol biosynthesis. Hyperglycemia leads to the consumption of more than 30% of glucose by producing sugar alcohols [76,98]. In addition, sugar alcohols may also originate from microbiota and food metabolism in the gut due to disrupted gut barrier function and bacterial translocation in liver disease [105] (Figure 8).

## 4. Materials and Methods 

### 4.1. Participants and Collection of Clinical Samples

Liver tissue and whole blood samples were obtained from 53 participants recruited between February 2011 and October 2012. Written informed consent was obtained from each participant. The study protocol (Code 00185/2019) was approved by the ethics committee of the National Liver Institute at Menoufia University, Menoufia, Egypt. Liver tissues were collected during surgery from 23 patients diagnosed with HCC. Two types of liver tissues were obtained from each patient as surgical specimens during the operation including HCC liver tissues and adjacent non-malignant liver tissues. Whole blood samples were obtained as venipuncture from 53 participants from the lower left arm including 15 healthy individuals, 15 CLD patients (HCV ± HBV infected subjects), and the same 23 patients who were diagnosed with HCC. Diagnosis of HCC was initially based on AFP > 200 ng/mL, ultrasound imaging and triphasic spiral computed tomography for focal lesions. This diagnosis was later confirmed histopathologically after surgical resection for all HCC cases.

All CLD cohort diagnosis was performed by PCR testing for hepatitis C virus infection and hepatitis B virus infection [106,107] clinically validated for positive chronic infection for more than 6 months. Secondly, all subjects were tested by diagnostic ultrasonography (U/S) examination to exclude cirrhosis. While the U/S test is operator dependent, it can still reliably exclude cirrhosis in combination with other laboratory tests that were performed for serum biochemical parameters, including bilirubin, albumin and prothrombin time. HCC diagnosis was confirmed histopathologically after resection. All blood samples underwent laboratory investigation for liver function tests: Alanine transaminase (ALT) and Aspartate transaminase (AST), Alpha-fetoprotein (AFP) were performed using a Beckman Coulter (Synchron CX 9 ALX) Clinical Auto analyzer (Beckman Instruments, Fullerton, CA, USA). Hemoglobin and platelet count were performed by a Coulter Counter T660 (Coulter Electronics, Hielaeh, Fl, USA). International Normalized ratio (INR) was calculated from prothrombin time measured by an STA-Stago Compact CT auto analyzer using reagents supplied by Dade-Behring. Tissue specimens and blood samples were stored at −80 °C prior to analysis. Samples were shipped on dry ice to the West Coast Metabolomics Center at UC Davis.

### 4.2. Sample Pretreatment

About 4 mg of frozen liver tissues were weighted and placed into a 1.5 mL Eppendorf tube, in addition to 3 small stainless-steel grinding balls. Samples were then placed into a GenoGrinder 2010 (Spex SamplePrep, Metuchen, NJ, USA) for 2 min at 1350 Hz. Homogenized tissue samples were subsequently prepared for metabolomic analyses in the same way as plasma samples.

Whole blood samples were aliquoted into 1.5 mL Eppendorf tubes, stored at −80 °C and shipped on dry ice. After thawing for 30 min at room temperature, samples were centrifuged for 30 min at 14,000× *g*. 20 µL of the supernatant was used for extraction. 1 mL of a degassed, −20 °C mixture of acetonitrile (ACN): isopropanol (IPA): water (H_2_O) (3:3:2, *v*/*v*/*v*) was added, samples were vortexed, shaken for 5 min and then centrifuged at 14,000× *g* for 2 min. 450 µL of supernatant was transferred to a new tube. After concentrating to complete dryness, the pellet was extracted with 500 µL 1:1 acetonitrile: water, vortexed and centrifuged for 2 min at 14,000× *g* to remove any residual proteins or triacylglycerides. The supernatant was concentrated to complete dryness, metabolites were derivatized by 10 µL of methoxyamine hydrochloride in pyridine (40 mg/mL) at 30 °C for 90 min, followed by trimethylsilylation of acidic protons using 90 µL of N-methyl-N-(trimethylsilyl) trifluoroacetamide (MSTFA, Sigma-Aldrich, St. Louis, MO, USA) for 30 min at 37 °C.

### 4.3. Metabolomics Data Acquisition and Data Processing

For retention time correction, C8–C30 fatty acid methyl esters (FAMEs) were added as internal standards. Samples were analyzed by a Leco Pegasus IV time of flight mass spectrometer using splitless injection into an Agilent gas chromatograph (for details, see [108]). Both plasma and tissue samples were randomized and analyzed along with quality control and method blank samples. Raw spectra for plasma and tissue metabolites were processed by BinBase software [40]. The BinBase algorithm matches each sample mass spectrum data and retention index against the FiehnLib mass spectral library [40] and the NIST17 commercial library. The reported metabolites were quantified using ion peak heights of deconvoluted unique ions and were normalized by the sum of all annotated metabolites intensity across the entire study.

### 4.4. Statistical Analysis

Statistical analysis of clinical and biochemical data was performed for two group-comparisons by *t*-tests. The Chi-square test was used to test the null hypothesis in the relation between two or more variables. For metabolite data, non-parametric univariate statistical tests were used to test for significantly altered compounds between the studied groups. Non-parametric paired Wilcoxon Signed Rank test was used for data from liver tissues. For blood metabolomics, Kruskal–Wallis and Mann–Whitney U tests were used to detect significant features among the three studied groups and between paired comparisons. Raw *p*-values < 0.05 and adjusted p-value using the Benjamini-Hochberg’s False Discovery Rate (FDR) q < 0.10 were considered significant. Statistical analyses were calculated using R software version 3.5.3 (R Foundation for Statistical Computing, Vienna, Austria) and MetaboAnalyst R 4.0 [109]. Multivariate and univariate statistical analyses were used for metabolomics data to detect groups of samples that align with clinical descriptors, and to detect groups of metabolites (“pathways”) that are regulated by the disease progression. Unsupervised multivariate analyses were used mainly for quality control to study unrelated factors such as machine drifts or hidden bias in analytical procedures. We used sparse partial least squares—discrimination analysis (sPLS-DA) as a multivariate statistical method for sparse data to reflect the use of a few samples in comparison to a high number of variables [110]. Heat map hierarchical clustering was performed to provide a graphical summary of differentially regulated metabolites, using a clustering Euclidian for distance measurement and ward clustering algorithm to assort data by similarity patterns. In addition, chemical set enrichment analysis was performed by ChemRICH software to highlight chemical classes that were significantly altered in HCC pathogenesis [42].

## 5. Conclusions

At current, HCC patients are diagnosed too late, delaying interventions and impairing therapeutic success. Focusing on primary metabolites, we here established clear blood-based metabolic differences between healthy controls, CLD patients and HCC patients that were reflected in altered metabolic phenotypes in early-stage HCC tumors and paired non-malignant liver tissue biopsies. Importantly, we solidified large differences in carbohydrate metabolism between these study groups and distinguished differences in liver metabolism of endogenous enzymes from the differential utilization of microbial and food metabolites. Our findings are consistent with earlier reports on the pathogenic role of aldo-keto reductases in HCC, possibly opening novel therapeutic options. In addition, we showed that screening for small panels of metabolites by GC-MS drastically improves HCC diagnosis in comparison to the classic biomarker alpha-fetoprotein. This finding may lead to new efforts to validate and solidify the accuracy of these biomarkers in HCC diagnosis and treatment.

## Figures and Tables

**Table 1:** Clinical and biochemical characteristics of study subjects.

Parameter	HCC	Chronic Liver Disease (CLD)	Healthy Control	*p*-Value
Number	23	15	15	
Gender (M/F)	17/6	11/4	10/5	0.877 ^(1)^
Age (Years) Mean (range)	53 (23–73)	46 (24–67)	50 (23–64)	0.311 ^(2)^
Alanine transaminase (IU/L) Mean (range)	56 (18–160)	58 (20–134)	27 (16–37)	0.878 ^(a)^ 0.003 ^(2)^
Aspartate transaminase (IU/L) Mean (range)	65 (21–195)	48 (20–119)	26 (18–35)	0.115 ^(a)^ 0.001 ^(2)^
Hemoglobin (g/L) Mean (range)	12.9 (9.3–16.7)	14 (11.9–16.6)	13 (11.8–14.3)	0.011 ^(a)^ 0.02 ^(2)^
Platelet count (10^3^/L) Mean (range)	193 (53–686)	240 (172–355)	217 (166–260)	0.158 ^(a)^ 0.370 ^(2)^
International normalized ratio Mean (range)	1.34 (1–2.8)	1.03 (0.9–1.14)	0.97 (0.9–1.1)	0.005 ^(a)^ 0.001 ^(2)^
current HCV infection (Y/N) ^(c)^	13/10	12/3	0/15	3.2 × 10^−5 (1)^ 0.13 ^(b)^
current HBV infection (Y/N) ^(c)^	2/21	3/12	0/15	0.18 ^(1)^ 0.25 ^(b)^
Alfa fetoprotein (AFP) (ng/mL) Mean (range)	268.3 (2.6–2000)	8.9 (1.53–60)	2.6 (1.8–3.5)	0.021 ^(a)^ 0.016 ^(2)^

^(a)^*t*-test between HCC and CLD patients. ^(b)^ Chi-square test between HCC and CLD patients. ^(c)^ Remaining eight HCC patients had recent HCV infection up to 2 years prior to surgery. ^(1)^ Chi-square test for healthy controls, HCC, CLD. ^(2)^ ANOVA test for healthy controls, HCC, CLD.

**Table 2:** Characteristics of hepatocellular carcinoma (HCC) patients and HCC tissues.

Age of the Patients (years) Mean (range)	53 (23–73)
Gender	
Male	17 (74%)
Female	6 (26%)
Tumor stage (TNM)	
I (Early)	7 (30%)
II (Early)	9 (39%)
III (Late)	5 (22%)
IV (Late)	2 (9%)
Focal Lesion	
Single	15 (65%)
Multiple	8 (35%)
Cirrhosis	
Yes	21 (91%)
No	2 (9%)
Tumor Size	
<5 cm	19 (83%)
>5 cm	4 (17%)

## Supplementary Materials

The following are available online at https://www.mdpi.com/2072-6694/12/2/484/s1. Supplementary S1: Table S1.A: Whole data set and compounds detected in blood using GCTOFMS, Table S1.B: Compounds related to the heatmap (Figure 1c), Table S1.C: Univariate statistics of significant blood compounds comparing healthy control versus CLD and comparing CLD versus HCC, Table S1.D: Detailed blood metabolite view on Hierarchical Cluster Analysis clusters that separate HCC, CLD, and healthy control subjects, Table S1.E: ChemRICH clusters and set enrichment significance for blood metabolites, Table S1.F: Univariate statistics comparing blood metabolites in CLD versus early HCC and comparing early-stage versus late-stage HCC, Supplementary S2: Table S2.A: Whole data set and compounds detected in tissues using GCTOFMS, Table S2.B: Univariate statistics of significant identified metabolites comparing HCC tumors to paired non-malignant liver tissues, Table S2.C: ChemRICH clusters and set enrichment significance for HCC tumor metabolites versus non-malignant tissues, Table S2.D: Univariate statistics comparing identified metabolites in early-stage and late-stage HCC tumors versus its paired non tumor tissues.
